# Ultrasound Features and Ultrasound Scores in the Differentiation between Benign and Malignant Adnexal Masses

**DOI:** 10.3390/diagnostics13132152

**Published:** 2023-06-23

**Authors:** Mar Pelayo, Javier Sancho-Sauco, Javier Sanchez-Zurdo, Leopoldo Abarca-Martinez, Carlota Borrero-Gonzalez, Jose Antonio Sainz-Bueno, Juan Luis Alcazar, Irene Pelayo-Delgado

**Affiliations:** 1 HM Puerta del Sur, HM Rivas Hospital, 3428521 Madrid, Spain; mar_pelayo@yahoo.es; 2 Department of Obstetrics and Gynecology, Universitary Hospital Ramón y Cajal, Alcalá de Henares University, 3428034 Madrid, Spain; jsanchosauco@gmail.com (J.S.-S.); leohrcfetal@gmail.com (L.A.-M.); 3 Insight Technology Solutions, 3428108 Madrid, Spain; javier.sanchez.zurdo@gmail.com; 4 Department of Obstetrics and Gynecology, Valme Universitary Hospital, 3441014 Seville, Spain; carlota_borrero@hotmail.com (C.B.-G.); jsainz@us.es (J.A.S.-B.); 5 Department of Obstetrics and Gynecology, Clínica Universidad de Navarra, 3431008 Pamplona, Spain; jlalcazar@unav.es

**Keywords:** transvaginal ultrasound, adnexal masses, IOTA simple rules risk assessment, O-RADS, ADNEX model, CA125, subjective assessment

## Abstract

Background: Several ultrasound (US) features help ultrasound experts in the classification of benign vs. malignant adnexal masses. US scores serve in this differentiation, but they all have misdiagnoses. The main objective of this study is to evaluate what ultrasound characteristics are associated with malignancy influencing ultrasound scores. Methods: This is a retrospective analysis of ultrasound features of adnexal lesions of women managed surgically. Ultrasound characteristics were analyzed, and masses were classified by subjective assessment of the ultrasonographer (SA) and other ultrasound scores (IOTA Simple Rules Risk Assessment SRRA, ADNEX model, and O-RADS). Results: Of a total of 187 adnexal masses studied, 134 were benign (71.7%) and 53 were malignant (28.3%). SA, IOTA SRRA, ADNEX model with or without CA125 and O-RADS had high levels of sensitivity (93.9%, 81.1%, 94.3%, 88.7%, 98.1%) but lower specificity (80.2%, 82.1%, 82.8%, 77.6%, 73.1%) with similar AUC (0.87, 0.87, 0.92, 0.90, 0.86). Ultrasound features significantly related with malignancy were the presence of irregular contour, absence of acoustic shadowing, vascularized solid areas, ≥1 papillae, vascularized septum, and moderate-severe ascites. Conclusion: IOTA SRRA, ADNEX model, and O-RADS can help in the classification of benign and malignant masses. Certain ultrasound characteristics studied in ultrasound scores are associated with malignancy.

## 1. Introduction

Performing a correct ultrasound (US) diagnostic approach to an adnexal mass is not a simple task due to the variety of diagnoses and the US characteristics are not always related exclusively with benignity or malignancy.

IOTA (International Ovarian Tumor Analysis) group previously published the terms, definitions, and measures that would serve to describe adnexal lesions in an attempt to homogenize and standardize criteria [1].

When describing an adnexal lesion, it is necessary to detail where it is located (right/left/Douglas/abdomen/pelvis), the size (three measures in two perpendicular planes), if it′s solid or cystic and the number of locules (uni/multilocular) according to which lesions may be cystic uni/multilocular, solid-cystic uni/multilocular, solid or unclassifiable [1]. In addition, it should be reported if it contains septum (band of tissue that crosses a cystic mass from its inner surface to its contralateral side), solid portion (echogenic tissue), papillary projections (solid projection in a cystic cavity ≥3 mm height), acoustic shadowing (hypoechoic bands behind a structure), or ascites (liquid outside the pouch of Douglas). The septa, solid parts or papillary projections may be examined with color Doppler to assess their degree of vascularization (score color 1: no color; score color 2: small flow; score color 3: moderate; score color 4: intense).

With the study of these characteristics, the subjective diagnosis by an experienced sonographer (Subjective Assessment) has the greatest diagnostic capacity when classifying adnexal masses [2,3,4]. However, US experts are not always available. 

In an attempt to help other less experienced sonographers, US scores were created trying to quantify the importance of each of the aforementioned characteristics and support the diagnosis objectively. IOTA Simple Rules [5] and the IOTA Simple Rules Risk Assessment were the first calculators to predict the risk of malignancy [6] which included items such as US examination at oncology center (yes/no) and certain US characteristics (absent/present) related to benignity (unilocular cyst, solid part <7 mm, acoustic shadowing, multilocular smooth mass <100 mm with no blood flow), or malignancy (irregular solid tumor, ascites, ≥4 papillae, multilocular irregular solid tumor ≥100 mm with high blood flow).

The ADNEX model (Assessment of Different Neoplasia in the Adnexa) was published by the IOTA group as another complementary score that calculate the probability of malignancy [7]. It includes the size of the lesion (mm), size of solid part (mm), number of locules (>10), number of papillae (non to more than 3), acoustic shadowing, and ascites. Other criteria include age, values of CA125 (UI/mL), and if the ultrasonographer works in a referral center. It has the option to subclassify malignant lesions according to staging (borderline, state I, state II–IV, metastatic) and to enter the values of CA125 to refine in such staging. 

Lastly, the American College of Radiology (ACR) introduced the O-RADS system (Ovarian Adnexal Reporting and Data System) [8] using a previously described lexicon [9] classifying adnexal masses into five categories according to their US appearance, including an associated probability of malignancy as well as a recommended management guide based on that risk. US items studied include the differentiation between cystic lesions (simple/non simple cyst, uni/multilocular cyst, smooth/irregular inner wall, smooth/irregular inner walls/septations) and solid ones (smooth/irregular contour), size, presence of solid component (number of papillary projections, size, score color 1–4), acoustic shadows, ascites, and peritoneal nodules. It also includes the definition of classic benign lesions (typical hemorrhagic cyst, dermoid cyst, endometrioma, periovarian cyst, peritoneal inclusion cyst, hydrosalpinx). 

The objective of this work is to differentiate which US features of the adnexal masses included in US scores are associated with benignity or malignancy.

## 2. Materials and Methods

This is a retrospective US analysis of adnexal masses in women operated in the last two years (January 2021 to December 2022) in the Gynecology Department of a University tertiary care hospital in Madrid (Spain).

**Figure 1:**
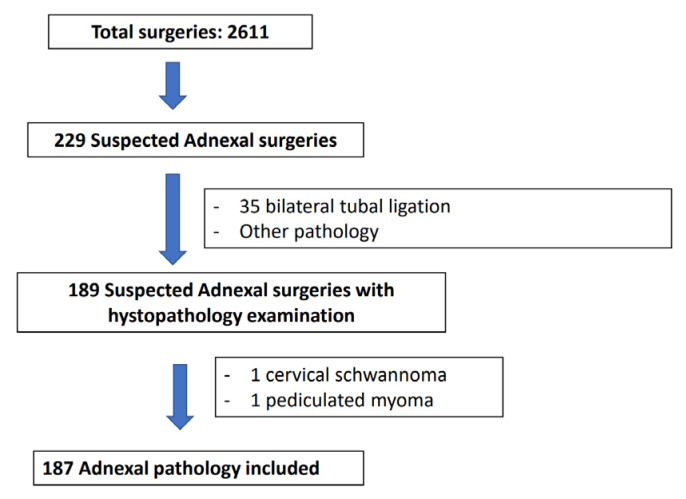
Flowchart of patients included in the present study.

Of a total of 2611 surgeries, 229 were referred for performing adnexal surgery (8.8%). Thirty-five cases of family planning bilateral tubal ligation and another five cases in which no adnexal tissue (i.e hemoperitoneum after ovarian puncture for in vitro fertilization, hemorragic corpus luteum) were excluded. In addition, in two patients, surgery did not confirm adnexal pathology. The rest of the lesions (n: 187) were included in the sample (Figure 1). A total of 30 patients were received by non-expert sonographers in out-patient clinic, 15 came from the emergency room, and 142 cases were studied by expert sonographers. Local clinical protocols were applied to decide patients’ management. Clinical information, US images, or US medical reports were reviewed. Approval was obtained from the Hospital Ethics Committee. The evaluation of indeterminate malignant masses evaluated exclusively by US experts have been published before [10]. 

### 2.1. Criteria for Inclusion and Exclusion

The women included had a US study performed 180 days or less before surgery, whose images/US medical report were saved in the hospital’s PACS (Picture Archiving and Communication System) or in the ultrasound equipment. Adnexal masses studied histologically by ambulatory transvaginal ecoguided biopsy were published elsewhere [11]. US medical report included US features described below. Histological study was conducted based on WHO criteria [12,13]. Masses with no available images or without quality criteria were not included. 

### 2.2. Methodology

Data included clinical information, values of CA125 (IU/mL), surgical approach and histopathological study.

Ultrasound equipment provided a RIC 5–9D 4–9 MHz endovaginal probe and a RAB6-D 2–8 MHz transabdominal probe Voluson E8 (GE Healthcare, US, Milwaukee, WI, USA), Canon Aplio A and Xario 100 (Canon Medical Systems corporation, Tokyo, Japan).

US features collected were largest size (mm), contour (regular/irregular), acoustic shadowing, presence of solid areas (mm) and their doppler color (score color 1–4), septum (characteristics, score color 1–4), number of locules (none/1/2–9/≥10), presence of papillae (number, size, score color 1–4), presence of ascites (no-mild/moderate-severe). US scans were performed by experienced or non-experienced sonographers all of them following the scanning methodology and the lexicon described by the IOTA group [1]. Images and clinical reports were stored in the US software, PACS and electronical clinical history. Two US gynecological experts classified images and reviewed clinical reports blind to histological diagnosis.

US scores included Subjective Assessment with a final classification in benign or malignant masses.

IOTA Simple Rules Risk Assessment (%) was calculated by accessing the website https://homes.esat.kuleuven.be/~sistawww/biomed/ssrisk/ (accessed on 1 March 2023).

The data of the ADNEX model with and without CA125 was obtained from the website https://www.iotagroup.org/sites/default/files/adnexmodel/IOTA%20-%20ADNEX%20model.html (accessed on 1 March 2023). Resulting parameters included % benign tumor, % malignancy, % borderline, % stage I, % stage II–IV, % metastatic.

O-RADS can be staged from 0–5. Classification of adnexal masses can be studied from https://www.acr.org/-/media/ACR/Files/RADS/O-RADS/US-v2022/O-RADS-v2022-Updates.pdf (accessed on 1 March 2023).

A cut-off point of 10% risk of malignancy was used to consider malignant masses (≥10%).

If lesions were bilateral, the most complex or the largest mass was chosen.

Levels of CA125 were analyzed by a Alinity i CA125 II Reagent Kit (Abbot, Chicago, IL, USA).

Pathology experts in gynecological pathology with more than 15 years of experience based their classification criteria in the guidelines of the World Health Organization [12]. Borderline tumors were included in the malignancy group. 

Data recording and basic statistics used Excel for Microsoft 365 MSO (64-bit version 2302) (Redmon, WA, USA). The Python programming language (v3.10.7) was used to process the information collected, as well as the Pandas data manipulation and processing libraries (v1.5.2), NumPy numerical processing (v1.23.1) and file processing and encryption with msoffcrypto-tool (v5.0.0). The development environment was Visual Studio Code (v1.77.0) and Spyder IDE (v5.3.3). PSPP (v1.6.2), equivalent to SPSS (proprietary software), was also used for data processing and analysis. Categorial variables are expressed as numbers and percentage, and comparisons were calculated with Pearson’s Chi-square test or Fisher’s exact test with significance level considered at <0.05. US scores models were evaluated with Excel and PSPP. Sample size was not estimated, and statistical power was not calculated.

## 3. Results

Basal characteristics are summarized in Table 1 and Table 2. Mean age was 49.6 years ± 15.9, showing higher values in patients with malignant (55.6 years ± 14.9) than benign disease (47.2 years ± 15.7, *p <* 0.001). Higher proportion of menopausal women was found in the malignancy group (67.9%, n: 36) than in the benign one (36.6%, n: 49, *p <* 0.001). Other features such as parity, BMI or clinical presentation did not show any significant difference. In relation to the type of surgery, laparotomy (67.9%, n: 36) and hysterectomy with double adnexectomy (43.4%, n: 23, *p <* 0.001) was more frequent in the approach of malignant masses. Also, CA125 values were increased in the malignant group (707.5 IU/mL ± 2154.5, *p <* 0.001).

Histological diagnosis of benign and malignant adnexal masses is detailed in Table 3.

US features of adnexal masses are summarized in Table 4.

Characteristics significatively associated with benignity are the presence of a regular contour (*p >* 0.001), acoustic shadows (benign: 72.4% n: 97; malignant: 22.6% n: 12, *p >* 0.001), absence or low Doppler color in septae (*p <* 0.001), absence of papillae (*p <* 0.001), small size of papillae (*p*: 0.005), and no or mild ascites (*p*: 0.025). 

**Figure 2:**
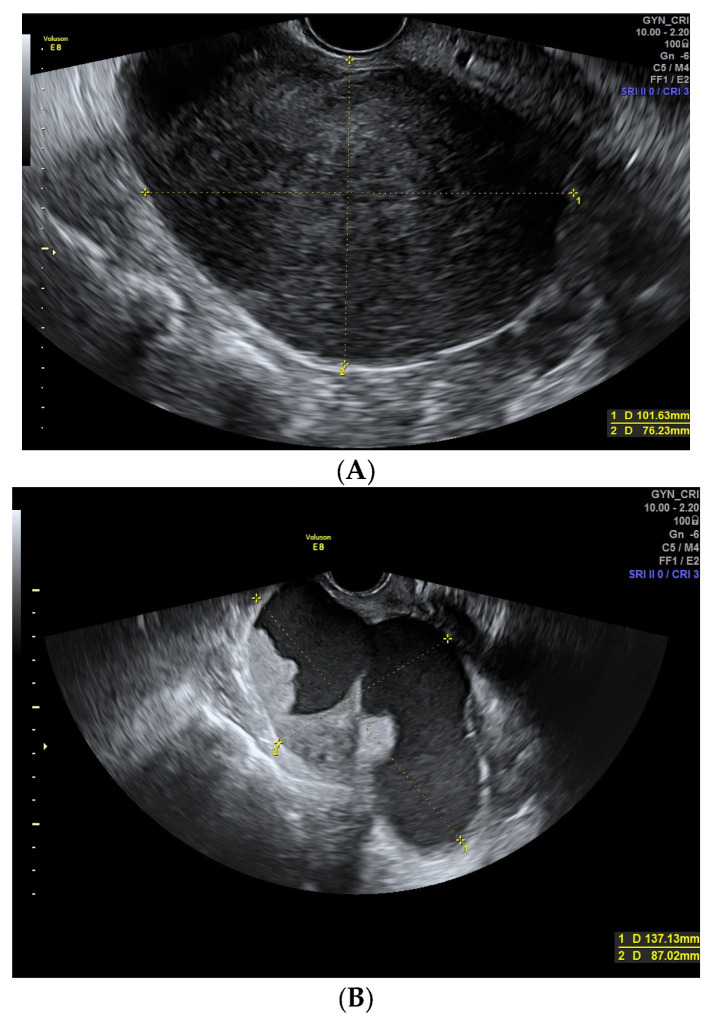

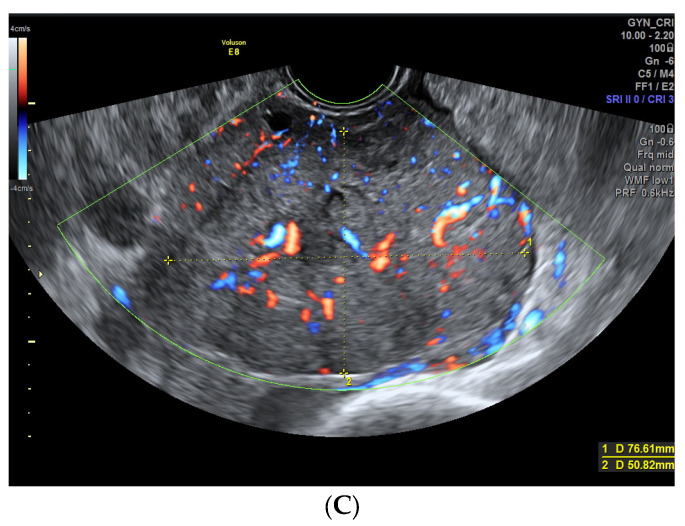
(**A**) Fibroma in a 59-year-old woman. A solid regular mass with smooth contour can be seen with scarce Doppler color (score color 2). (**B**,**C**) Malignant ovarian lesions with irregular contour are shown. Image (**B**) shows a cystic mass with an irregular internal contour corresponding to a 55-year-old woman with a clear cell carcinoma. Image (**C**) shows a solid mass with moderate-intense Doppler color (score 3–4) with an irregular external contour, corresponding to a serous ovarian carcinoma in a 64-year-old woman.

**Figure 3:**
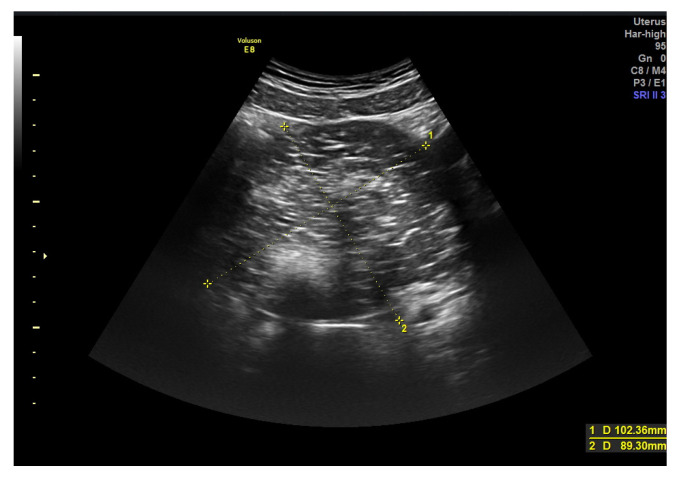
Teratoma in a 33-year-old woman. Image shows an heterogeneous regular mass seen with abdominal probe. Intralesional acoustic shadows can be seen.

**Figure 4:**
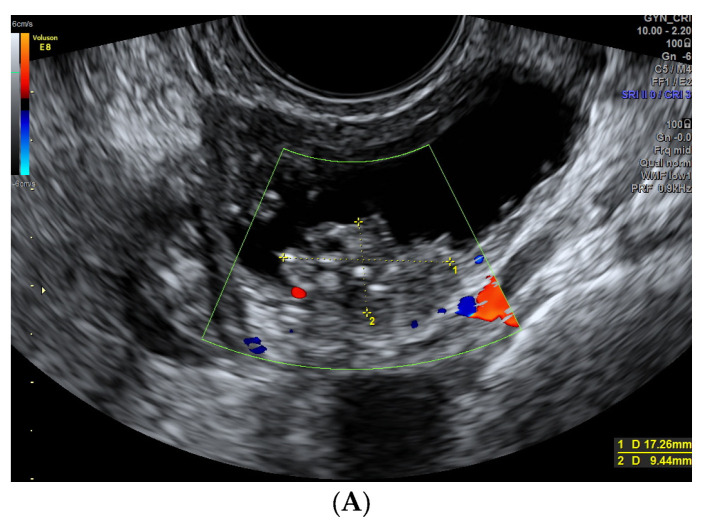

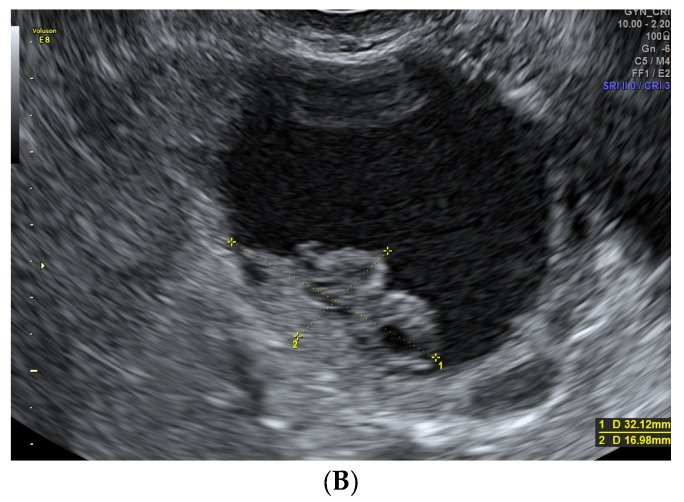
*(***A**) Cystoadenofibroma in a 62-year-old woman showing unilocular solid cyst with a papillary structure. (**B**) A 49-year-old woman with a borderline serous carcinoma showing a nonvascularized papillae. Note the similarity between both images.

Characteristics significatively associated with malignancy are the presence of an irregular contour (benign: 9.0% n: 12 vs. malignant: 50.9% n: 27, *p* > 0.001) (Figure 2), absence of acoustic shadows (benign: 27.6% n: 37 vs. malignant: 77.4% n: 41, *p >* 0.001) (Figure 3), presence of solid areas (benign: 25.4% n: 34 vs. malignant: 88.7% n: 47, *p <* 0.001) (Figure 2), doppler of solid areas (score color 3–4 in benign masses: 20.0% n: 7 vs. malignant: 70.2% n: 33, *p <* 0.001), moderate or high Doppler color in septae (score color 3–4 in benign masses: 10.0% n: 5 vs. malignant: 52.2% n: 12, *p <* 0.001), presence of papillae (> 1 papillae benign: 6.0% n: 8, vs. malignant:, 24.5% n: 13, *p <* 0.001) (Figure 4), bigger size of papillae (benign:13.7 ± 7.9 mm; range: 4.0–39.0 mm vs. malignant: 29.8 ± 19.8 mm, range: 3.3–90 mm, *p*: 0.005) and moderate or severe ascites (benign: 5.2% n: 7 vs. malignant: 15.1% n: 8, *p*: 0.025).

**Figure 5:**
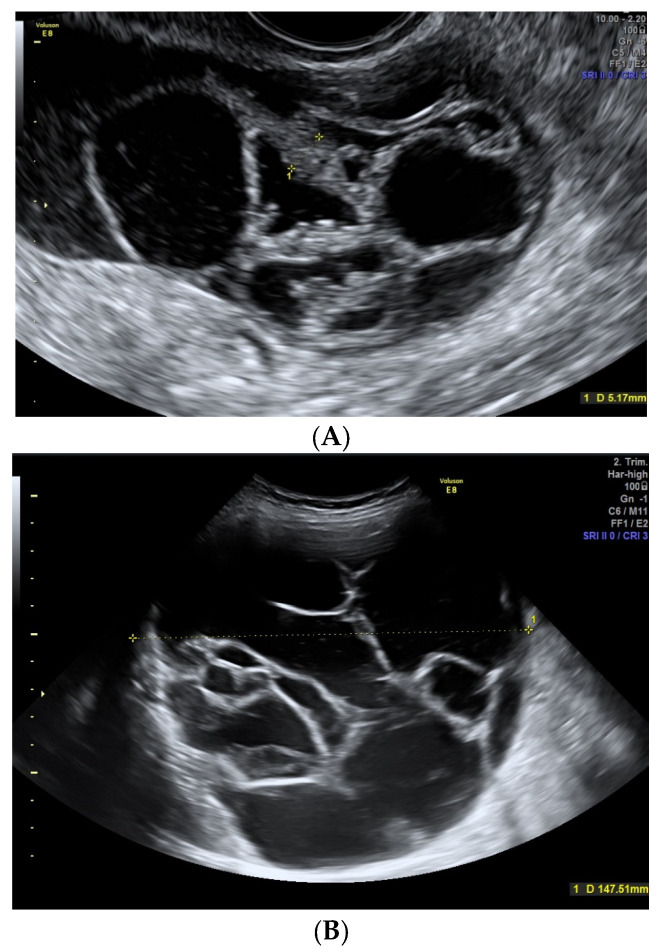
(**A**) Ultrasound images of a 43-year-old woman with a mucinous cystoadenoma. It shows a multilocular cyst with thick and irregular septae. (**B**) A 78-year-old woman with a Brenner Tumor. A big multicystic lesion is observed with thin avascular septum.

No significant differences were found between benign and malignant masses in: largest size of the lesion (benign: 83.1 ± 45.9 mm vs. malignant: 101.9 ± 49.1 mm, *p*: 0.128), size of solid areas (benign: 47.9 ± 35.2 mm vs. malignant: 53.4 ± 41.8 mm, *p*: 0.541), presence of thick or irregular septum (benign: 8.2%, n: 11 vs. malignant: 19.2%, n: 10, *p*: 0.078) (Figure 5), number of locules (*p*: 0.288) or doppler in papillae (*p*: 0.071).

Diagnostic performance of the different methods of classification of US masses are summarized in Table 5. Sensitivity, Specificity, Positive Predictive Value, and Negative Predictive Value for Subjective Assessment was 93.9% (87.4–100), 80.2% (74.1–86.3), 63.9%, 97.2%, respectively; for SRRA: 81.1% (71.6–90.6), 82.1% (76.2–88.0), 64.2% and 91.7%, respectively; for ADNEX model with CA125: 94.3% (88.2–100), 82.8% (77.0–88.6), 68.5%, 97.4%, respectively; ADNEX model without CA125: 88.7% (80.7–96.7), 77.6% (71.4–83.8), 61.0% and 94.5%, respectively; for O-RADS: 98.1% (94.5–100), 73.1% (66.7–79.5), 59.1%, and 99.0%, respectively. 

**Figure 6:**
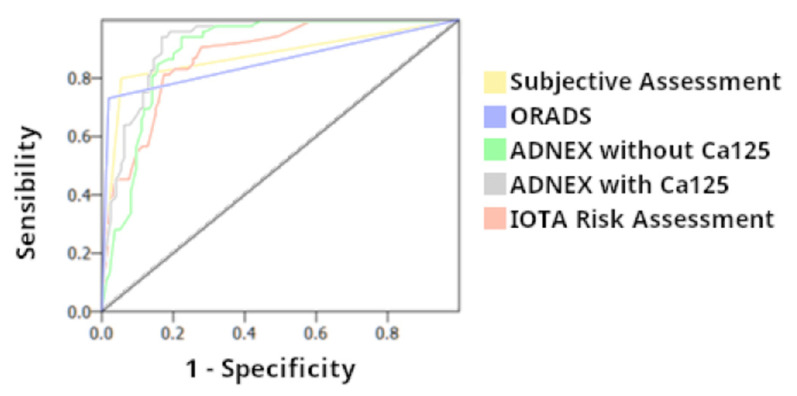
Diagnostic performance in the AUC (Area Under Curve) of the Subjective Assessment, IOTA Simple Rules (SR), IOTA Simple Rules Risk Assessment, O-RADS, and ADNEX model with and without CA125.

AUC and OR for Subjective Assessment, SRRA, ADNEX model with/without CA125, and O-RADS was 0.87, 0.87, 0.92, 0.90, and 0.86, respectively, and 61.9, 19.7, 80.43, 27.2, and 141.6 (Figure 6).

## 4. Discussion

Incorrect classification of an adnexal lesion as malignant or benign can lead to under or over diagnose adnexal cancers. Considering a benign lesion as malignant can lead to a wrong referral to specialized gynecological oncology center, forcing surgical indications and other medical tests, generating anxiety and concern in women. Instead, the missing diagnose of an ovarian tumor will leave women untreated and delay the definitive treatment, assuming the progression of the disease which will worsen the prognosis particularly in ovarian cancers [14]. Advantages of classifying correctly adnexal masses includes helping the management of the patient assessing if surgery is necessary, determining the type of surgery, laparoscopic or laparotomic approach, complete with additional image studies (CT, MRI) or request for tumor markers. In addition, an early diagnosis increases the survival rate, offering correct follow-up and treatment at the lowest cost [4].

In our study certain US features are related with malignancy (Table 4). 

The size of the tumor has been traditionally related to malignancy. In our series, there is no statistically significant difference from the average size of benign or malignant lesions. This could be explained by the selection of the patients, since the sample was taken of patients with adnexal lesions with indications of surgery and small lesions considered benign are less likely to be surgically removed. Also, there was a wide range of sizes of lesions removed (benign: 83.1 mm (±45.9; 23.0–289.0; malignant: 101.9 mm (±49.1; 21.0–220.0). Di Legge et al. [15] analyzed 2445 patients with one or more adnexal mass and grouped the lesions according to their largest diameter: either smaller than 4 cm (small lesion), between 4–10 cm (medium) or 10 cm (large tumors) with a probability of malignancy of 10%, 19% and 40% in each group. In our series we found 20 lesions (10.7%) of small size (<4 cm), 108 (57.8%) of intermediate size (4–10 cm) and 59 (31.5%) large (>10 cm) with a malignancy percentage of 15.0%, 25.9%, and 37.3%, respectively, in agreement with the results of Di Legge. In another study [16] of 129 surgically small lesions (≤ 2.5 cm), 12% were found to be borderline and 8% invasive tumors. In our series we had five cases ≤ 2.5 cm and one case was malignant (sex cordon tumor) and four benign (cystoadenofibroma, serous cystadenoma, endometriosis, bilateral hypertecosis). Bruno et al. [17] described a case of a small Leydig tumor (22 mm) that could be suspected by US. Another study estimated how US score models could differentiate malignant masses of different sizes [15]. They realized that small tumors were detected with lower sensitivity (56–84%) but higher specificity (83–96%). We can conclude then that although larger lesions are more likely to be malignant, not all large lesions must be malignant. 

Irregular contour was more frequently seen in malignant tumors. When the lesion is cystic it´s important to look at the internal wall to discard the existence of a solid papillary projection that would make it irregular [1]. In the cases of solid masses, the outer wall should be examined looking for irregularities (Figure 2). 

The presence of acoustic shadows is associated with benignity, although they may be seen in some malignant masses. Hack et al. [18] studied how O-RADS score could be modified by incorporating acoustic shadowing. He concluded that the addition of acoustic shadowing, considered a benign feature, improved the detection rate of O-RADS. In our series the presence of acoustic shadowing in malignant cases corresponded to five serous borderline tumors, four serous carcinomas three of them with largest size more than 10 cm, and other more infrequent cases. This could be explained by the fact that false images of acoustic shadows may appear in largest lesions, or due to calcifications inside the tumoral mass such as in mature cystic teratomas (Figure 3) [19].

Most malignant adnexal masses contain highly vascularized solid portions [20] as it is described in our series (*p <* 0.001). However, mean size of the solid areas is not significantly different. Among other reasons, 11 solid-looking fibroids are included in our sample, all of them with a solid area diameter greater than 4 cm and even in 5 cases greater than 8 cm, bigger than found by other authors [21,22] (Figure 2). Valentin et al. included fibromas as one of the most difficult diagnostic US diagnosis [23,24]. We also had seven other benign lesions with a solid portion > 10 cm that were considered suspicious in the subjective diagnosis that corresponded with two teratomas, two Brenner tumors, one cystoadenofibroma, a mucinous cystoadenoma, and an atypical endometrioma.

The presence of thick or irregular septum is traditionally associated with malignancy. In our series even if there is no difference, the vascularization of the septae (score color 3–4) is related to malignancy (*p <* 0.001). We found four cases of cystadenoma (Figure 5) with irregular septum, (two mucinous cystadenoma, two serous cystadenoma), one cystadenofibroma and two dermoid cysts. In our series, as described in literature [25], the most common presentation of cystadenomas was as uni-bilocular lesions (75%, 9/12) and only one case had more than ten locules. Also Pascual et al. [26] found that benign mucinous cystadenoma can also have solid components and/or moderate/intense vascularization, making it difficult to differentiate from borderline and other malignant masses. 

Regarding the number of locules, we did not find any significant relationship between having one or more than ten locules with malignancy. IOTA terminology describes unilocular cysts as those that do not contain solid parts or papillae and solid-cystic unilocular cyst as one that contains a solid part or at least a papilla [1]. If we analyze unilocular cystic tumors, none of them were malignant according to the results found by Valentin et al. [27] who calculated a probability of 0.96% of malignancy in unilocular cystic lesions. In contrast, solid-cystic unilocular cysts were found in 58.5% (31/53) cases of malignant masses. On the other hand, 7 adnexal masses with 8 to 10 locules corresponded to benign formations (3 cystadenofybroma, 2 Brenner tumor, 1 serous, and 1 mucinous cystadenoma). Brenner tumors can be solid or multicystic [28], as in our cases (Figure 5). Moro et al. [29] related >10 locules with borderline mucinous tumors. 

Solid papillary projections are considered as solid projections in the cyst cavity ≥ 3 mm [1]. In previous studies, papillary projections are considered typical US features of borderline and malignant serous tumors in not advanced stages [20,30]. In our sample, 36 lesions had at least 1 papilla. The lesions in which we found only one papillae (n: 15, 8.0%) were benign lesions (n: 7, 5.2%): two mucinous cystoadenoma, two cystoadenofibroma, one endometriosis, one functional cyst, one twisted cyst, all of them avascular (except one). US findings of more than one papillae were associated with malignancy (>1 papillae n: 13, 24.5%, *p* < 0.001). Two or more papillary projections (n: 21) were found in malignant masses (n: 13) most of them with vascularization (n: 8), and in eight cases of benign lesions, five of them cystoadenofibromas (Figure 4*).* Cystadenofibromas are sometimes difficult to classify [23,24,31] as they can appear most commonly as a unilocular solid cyst with papillary projections [32,33,34]. Other authors suggested that the presence of acoustic shadows behind a papillae can distinguish cystadenofibromas from other malignant masses, specially borderline tumors [16,33]. Of the 13 cases of borderline carcinoma found in our series, eight had at least one papillae (number of papillae: 1–10), of sizes ranging 12–45 mm, and three of them were vascularized (score color 3). The preoperative detection of borderline ovarian tumors is an important issue taking into account that young women with unfulfilled reproductive desires could benefit from fertility-preserving surgery as opposed to being diagnosed malignant tumors in which surgery is usually much more aggressive [35]. Ludovisi et al. [36] described the Serous Surface Papillary Borderline Ovarian Tumors, typically located in the ovarian surface, seen as irregular solid lesions surrounding normal ovarian parenchima. Landolfo et al. [37] studied 204 masses (20.6% borderline, 14.7% malignant), and found that the size of the papillae and blood flow were factors associated with malignancy. Also, in our series bigger sizes of papillae were found in malignant masses (*p*: 0.005). Fagotti et al. [38] suggested that if there was a solid portion >14 mm, papillae were vascularized or both, it would be more likely a malignant invasive lesion. We found that all malignant masses (except in two cases), showed vascularization in the papillae and moderate-high vascularity (score color 3–4) was more frequent in malignant masses although not statistically significant probably due to the limited sample size.

Moderate-severe ascites was found in seven cases (5.2%) with benign adnexal masses, all of them corresponding to ovarian fibromas as in Meigs´ syndrome [39] (Figure 2) and in eight malignant ones (15.1%), showing significant difference between both groups. However, our series could be biased since some of the adnexal tumors in advanced stages (for example, peritoneal carcinomatosis) benefited from a transvaginal US-guided biopsy [11] instead of surgical biopsy and were treated initially with neoadjuvant chemotherapy prior to surgery. Two cases of free liquid in the pelvis corresponded to blood in relation to a ruptured endometrioma.

Comparison of the different US scores (SA, SRRA, ADNEX model with and without CA125 and O-RADS) in the classification of benign and malignant masses have already been published by our group [10]. We included 122 patients who were referred to an expert sonographer for assessment of an adnexal mass of difficult characterization. In contrast, in the present article, up to 187 patients have been evaluated, including another 65 easily categorized adnexal masses that were initially evaluated by non-expert sonographers that shared the same US protocol, which allowed a later classification by US experts. That could be one of the reasons why sensitivity and specificity values improve in this publication by including masses not so difficult to classify. Thus, previous values of SA, SRRA, ADNEX models with and without CA125 and O-RADS values of sensitivity and specificity (87.8%, 78.1%, 95.1%, 87.8%, 90.2% and 69.1%, 72.8%, 74.1% 67.9%, 60.5%) improved in this sample in most of the scores evaluated (93.9%, 81.1%, 94.3%, 88.7%, 98.1% and 80.2%, 82.1%, 82.8%, 77.6%, 73.1%) (Table 5). Other studies have also shown good sensitivity in the use of US scores regardless of the degree of US experience [3]. High levels of sensitivity have been published for O-RADS (100–88%) [10,18,40,41,42,43,44] and ADNEX model (97–87%) [10,40,41,42,44], similar to the data obtained in the present study.

## 5. Limitations

Limitations of this work include small sample size. In addition, patients without surgical indication due to most probably benign lesions were not included in the study which could further increase the sensitivity of the scores. Besides, the US was performed in a single center with a unified protocol in the description of the adnexal masses which can differ from those followed by other institutions, diminishing its reproducibility but decreasing the retrospective study bias. 

## 6. Future Research Directions

None of the US scores have demonstrated to take into account all of the US features described in adnexal masses. More studies will have to quantify the value of the combination of these characteristics to try to establish more accurately the diagnosis of malignancy.

## 7. Conclusions

US features such as irregular contour, absence of acoustic shadowing, vascularized solid areas, ≥1 papillae, vascularized septum and moderate-severe ascites are related to malignancy. IOTA SRRA, ADNEX model and O-RADS can help in the classification of benign and malignant masses. 

## Figures and Tables

**Table 1:** Baseline conditions in the total group of patients and divided by histopathological study (1) Mann-Whitney test (2) Chi^2^ Pearson test /Fisher test (3) T student.

	Total (n: 187)	Benign (n: 134, 71.7%)	Malignant (n: 53, 28.3%)	*p* Value
Age (years)	49.6 (±15.9)	47.2 (±15.7)	55.6 (±14.9)	(1) 0.001
Menopause:				(2) <0.001
- Yes	85 (45.5%)	49 (36.6%)	36 (67.9%)	
- No	102 (54.5%)	85 (63.4%)	17 (32.1%)	
Parity:				(2) 0.371
- Nuliparous	91 (48.7%)	63 (47.0%)	28 (52.8%)	
- Parous	96 (51.3%)	71 (53.0%)	25 (47.2%)	
BMI (kg/m)	26.4 (±5.4) (n: 80)	27.1 (±5.3) (n: 52)	25.2 (±5.2) (n: 28)	(3) 0.130
Clinic:				(2) 0.114
- Asymptomatic	71 (38.0%)	56 (41.8%)	15 (28.3%)	
- Digestive	73 (39.0%)	53 (39.5%)	20 (37.7%)	
- Bleeding	16 (8.6%)	10 (7.5%)	6 (11.3%)	
- Other	27 (14.4%)	15 (11.2%)	12 (22.7%)	

(1) Man-Whitney test; (2) Chi2 Pearson/Fisher test; (3) T student.

**Table 2:** Surgical approach and CA125 levels in the total group of patients and divided by histopathological study.

	Total (n: 187)	Benign (n: 134)	Malignant (n: 53)	*p* Value
Surgical approach:				(2) <0.001
- Laparoscopic	125 (66.8%)	108 (80.6%)	17 (32.1%)	
- Laparotomy	62 (33.2%)	26 (19.4%)	36 (67.9%)	
Surgical procedure:				(2) <0.001
- Double adnexectomy	54 (28.9%)	39 (29.1%)	15 (28.3%)	
- Hysterectomy and double adnexectomy	34 (18.2%)	11 (8.2%)	23 (43.4%)	
- Cystectomy/ooforectomy/unilateral adnexectomy	99 (52.9%)	84 (62.7%)	15 (28.3%)	
Laterality:				(2) 0.238
- Right	78 (41.7%)	61 (45.5%)	17 (32.1%)	
- Left	59 (31.6%)	39 (29.1%)	20 (37.7%)	
- Bilateral	50 (26.7%)	34 (25.4%)	16 (30.2%)	
CA125 (IU/mL)	226.3 (±1181.0)	36.0 (±84.2)	707.5 (±2154.5)	(1) <0.001

(1) Man-Whitney test; (2) Chi2 Pearson/Fisher test.

**Table 3:** Histological diagnosis of benign/malignant adnexal masses.

	Total		Total
BENIGN	134	MALIGNANT	53
Mature cystic teratoma	29	Ovarian Serous carcinoma	19
Endometriosis	24	Clear cell carcinoma	9
Fibroma/Fibrotecoma	13	Serous Borderline carcinoma	8
Serous Cistoadenoma	19	Mucinous Borderline carcinoma	5
Mucinous Cistoadenoma	12	Endometrioid carcinoma	4
Cistoadenofibroma	10	Neuroendocrine carcinoma	1
Brenner Tumor	5	Stromal hyperplasia	1
Hidrosalpinx	6	Steroid cells carcinoma	1
PID	4	Disgerminoma	1
Functional cyst	2	Struma ovarii	1
Paraovarian cyst	4	Sex cord tumor	1
Hiperthecosis	1		
Twisted cyst	5		

PID: Pelvic Inflammatory Disease.

**Table 4:** US features in the total group of patients divided by histopathological study.

	Total (n: 187)	Benign (n: 134)	Malignant (n: 53)	*p* Value
Largest size (mm)	88.4 (±47.3)	83.1 (±45.9)	101.9 (±49.1)	(1) 0.128
Contour:				(2) <0.001
- Regular	148 (79.1%)	122 (91.0%)	26 (49.1%)	
- Irregular	39 (20.9 %)	12 (9.0%)	27 (50.9%)	
Acoustic shadowing:				(2) <0.001
- Yes	109 (58.3%)	97 (72.4%)	12 (22.6%)	
- No	78 (41.7%)	37 (27.6%)	41 (77.4%)	
Presence of solid areas:				(2) <0.001
- Yes	81 (43.3%)	34 (25.4%)	47 (88.7%)	
- No	106 (56.7%)	100 (74.6%)	6 (11.3%)	
Size of solid areas (mm)	51.1 (±39.1) (n: 81)	47.9 (±35.2) (n: 34)	53.4 (±41.8) (n: 47)	(3) 0.541
Doppler of solid areas (Score color):				(2) <0.001
- 1–2	42 (51.2%)	28 (80.0%)	14 (29.8%)	
- 3–4	40 (48.8%)	7 (20.0%)	33 (70.2%)	
Septum:				(2) 0.078
- None	118 (63.4%)	86 (63.2%)	32 (61.6%)	
- Thin	47 (25.3%)	37 (27.6%)	10 (19.2%)	
- Thick/Irregular	21 (11.3%)	11 (8.2%)	10 (19.2%)	
Doppler of septum (Score color):				(2) <0.001
- 1–2	56 (77.8%)	45 (90.0%)	11 (47.8%)	
- 3–4	16 (22.2%)	5 (10.0%)	12 (52.2%)	
Number of locules:				(2) 0.288
- 0 (solid mass)	2 (1.1%)	1 (0.7%)	1 (1.9%)	
- 1	127 (67.9%)	96 (71.6%)	31 (58.5%)	
- 2–9	47 (25.1%)	31 (23.1%)	16 (30.2%)	
- ≥ 10	11 (5.9%)	6 (4.5%)	5 (9.4%)	
Number of papillae:				(2) <0.001
- 0	151 (80.7%)	119 (88.8%)	32 (60.4%)	
- 1	15 (8.0%)	7 (5.2%)	8 (15.1%)	
- >1	21 (11.2%)	8 (6.0%)	13 (24.5%)	
Size papillae (mm):	23.1 (±17.7) (n: 36)	13.7 (±7.9) (n: 15)	29.8 (±19.8) (n: 21)	(3) 0.005
Doppler of papillae (Score color):				(2) 0.071
- 1–2	26 (70.3%)	14 (87.5%)	12 (57.1%)	
- 3–4	11 (29.7%)	2 (12.5%)	9 (42.9%)	
Ascites:				(2) 0.025
- No-mild	172 (92.0%)	127 (94.8%)	45 (84.9%)	
- Moderate-Severe	15 (8.0%)	7 (5.2%)	8 (15.1%)	

(1) Mann-Whitney test (2) Chi2 Pearson test/Fisher test (3) T student.

**Table 5:** Diagnostic performance of the Subjective Assessment of Adnexal Masses and other US scores: IOTA Simple Rules Risk Assessment, ADNEX model with and without CA125 and O-RADS. Values in parenthesis are 95% CI.

	Sensitivity (%)	Specifity (%)	PPV (%)	NPV (%)	AUC	OR
Subjective assesment	93.9 (87.4–100)	80.2 (74.1–86.3)	63.9	97.2	0.87	61.9
Simple Rules Risk Assesment	81.1 (71.6–90.6)	82.1 (76.2–88.0)	64.2	91.7	0.87	19.7
ADNEX model with CA125	94.3 (88.2–100)	82.8 (77.0–88.6)	68.5	97.4	0.92	80.43
ADNEX model without CA125	88.7 (80.7–96.7)	77.6 (71.4–83.8)	61.0	94.5	0.90	27.2
O-RADS	98.1 (94.5–100)	73.1 (66.7–79.5)	59.1	99.0	0.86	141.6

## Data Availability

Data are available upon reasonable request.

## Summary Statement

US scores (IOTA SRRA, ADNEX model and O-RADS) based in US features are good diagnostic tools in the classification of benign and malignant adnexal masses.

## Key Results

- US scores (IOTA SRRA, ADNEX model and O-RADS) have high sensitivity classifying benign and malignant adnexal masses
- There are certain US features related to malignancy (irregular contour, absence of acoustic shadowing, vascularized solid areas, ≥1 papillae, vascularized septum, moderate-severe ascites)

## Abbreviations

US	Ultrasound
SA	Subjective Assessment
IOTA	International Ovarian Tumor Analysis
SRRA	Simple Rules Risk Assessment
ADNEX model	Assessment of Different Neoplasias in the Adnexa
O-RADS	Ovarian Adnexal Reporting and Data System

## References

[1] Timmerman D., Valentin L., Bourne T.H., Collins W.P., Verrelst H., Vergote I. (2000). Terms, definitions and measurements to describe the sonographic features of adnexal tumors: A consensus opinion from the International Ovarian Tumor Analysis (IOTA) group. Ultrasound Obstet. Gynecol..

[2] Viora E., Piovano E., Poma C.B., Cotrino I., Castiglione A., Cavallero C., Sciarrone A., Bastonero S., Iskra L., Zola P. (2020). The ADNEX model to triage adnexal masses: An external validation study and comparison with the IOTA two-step strategy and subjective assessment by an experienced ultrasound operator. Eur. J. Obstet. Gynecol. Reprod. Biol..

[3] Tavoraitė I., Kronlachner L., Opolskienė G., Bartkevičienė D. (2021). Ultrasound Assessment of Adnexal Pathology: Standardized Methods and Different Levels of Experience. Medicina.

[4] Jeong S.Y., Park B.K., Lee Y.Y., Kim T.-J. (2020). Validation of IOTA-ADNEX Model in Discriminating Characteristics of Adnexal Masses: A Comparison with Subjective Assessment. J. Clin. Med..

[5] Timmerman D., Testa A.C., Bourne T., Ameye L., Jurkovic D., Van Holsbeke C., Paladini D., Van Calster B., Vergote I., Van Huffel S. (2008). Simple ultrasound-based rules for the diagnosis of ovarian cancer. Ultrasound Obstet. Gynecol..

[6] Timmerman D., Van Calster B., Testa A., Savelli L., Fischerova D., Froyman W., Wynants L., Van Holsbeke C., Epstein E., Franchi D. (2016). Predicting the risk of malignancy in adnexal masses based on the Simple Rules from the International Ovarian Tumor Analysis group. Am. J. Obstet. Gynecol..

[7] Van Calster B., Van Hoorde K., Valentin L., Testa A.C., Fischerova D., Van Holsbeke C., Savelli L., Franchi D., Epstein E., Kaijser J. (2014). Evaluating the risk of ovarian cancer before surgery using the ADNEX model to differentiate between benign, borderline, early and advanced stage invasive, and secondary metastatic tumours: Prospective multicentre diagnostic study. BMJ.

[8] Andreotti R.F., Timmerman D., Strachowski L.M., Froyman W., Benacerraf B.R., Bennett G.L., Bourne T., Brown D.L., Coleman B.G., Frates M.C. (2020). O-RADS US Risk Stratification and Management System: A Consensus Guideline from the ACR Ovarian-Adnexal Reporting and Data System Committee. Radiology.

[9] Andreotti R.F., Timmerman D., Benacerraf B.R., Bennett G.L., Bourne T., Brown D.L., Coleman B.G., Frates M.C., Froyman W., Goldstein S.R. (2018). Ovarian-Adnexal Reporting Lexicon for Ultrasound: A White Paper of the ACR Ovarian-Adnexal Reporting and Data System Committee. J. Am. Coll. Radiol..

[10] Pelayo M., Pelayo-Delgado I., Sancho-Sauco J., Sanchez-Zurdo J., Abarca-Martinez L., Corraliza-Galán V., Martin-Gromaz C., Pablos-Antona M.J., Zurita-Calvo J., Alcázar J.L. (2023). Comparison of Ultrasound Scores in Differentiating between Benign and Malignant Adnexal Masses. Diagnostics.

[11] Pelayo-Delgado I., Sancho J., Pelayo M., Corraliza V., Perez-Mies B., Del Valle C., Abarca L., Pablos M.J., Martin-Gromaz C., Pérez-Vidal J.R. (2023). Contribution of Outpatient Ultrasound Transvaginal Biopsy and Puncture in the Diagnosis and Treatment of Pelvic Lesions: A Bicenter Study. Diagnostics.

[12] WHO Classification of Tumours Editorial Board (2020). Female genital tumours. World Health Organization Classification of Tumours.

[13] McCluggage W.G., Singh N., Gilks C.B. (2022). Key changes to the World Health Organization (WHO) classification of female genital tumours introduced in the 5th edition (2020). Histopathology.

[14] Nasioudis D., Mastroyannis S.A., Ko E.M., Haggerty A.F., Cory L., Giuntoli R.L., Kim S.H., Morgan M.A., Latif N.A. (2022). Delay in adjuvant chemotherapy administration for patients with FIGO stage I epithelial ovarian carcinoma is associated with worse survival; an analysis of the National Cancer Database. Gynecol. Oncol..

[15] Di Legge A., Testa A.C., Ameye L., Van Calster B., Lissoni A., Leone F., Savelli L., Franchi D., Czekierdowski A., Trio D. (2012). Lesion size affects diagnostic performance of IOTA logistic regression models, IOTA simple rules and risk of malignancy index in discriminating between benign and malignant adnexal masses. Ultrasound Obstet. Gynecol..

[16] Di Legge A., Pollastri P., Mancari R., Ludovisi M., Mascilini F., Franchi D., Jurkovic D., Coccia M.E., Timmerman D., Scambia G. (2017). Clinical and ultrasound characteristics of surgically removed adnexal lesions with largest diameter ≤2.5 cm: A pictorial essay. Ultrasound Obstet. Gynecol..

[17] Bruno M., Capanna G., Di Florio C., Sollima L., Guido M., Ludovisi M. (2023). Sonographic characteristics of ovarian Leydig cell tumor. Ultrasound Obstet. Gynecol..

[18] Hack K., Gandhi N., Bouchard-Fortier G., Chawla T.P., Ferguson S.E., Li S., Kahn D., Tyrrell P.N., Glanc P. (2022). External Validation of O-RADS US Risk Stratification and Management System. Radiology.

[19] Heremans R., Valentin L., Sladkevicius P., Timmerman S., Moro F., Van Holsbeke C., Epstein E., Testa A.C., Timmerman D., Froyman W. (2022). Imaging in gynecological disease (24): Clinical and ultrasound characteristics of ovarian mature cystic teratomas. Ultrasound Obstet. Gynecol..

[20] Valentin L., Ameye L., Testa A., Lécuru F., Bernard J.-P., Paladini D., Van Huffel S., Timmerman D. (2006). Ultrasound characteristics of different types of adnexal malignancies. Gynecol. Oncol..

[21] Chen H., Liu Y., Shen L.-F., Jiang M.-J., Yang Z.-F., Fang G.-P. (2016). Ovarian thecoma-fibroma groups: Clinical and sonographic features with pathological comparison. J. Ovarian Res..

[22] Paladini D., Testa A., Van Holsbeke C., Mancari R., Timmerman D., Valentin L. (2009). Imaging in gynecological disease (5): Clinical and ultrasound characteristics in fibroma and fibrothecoma of the ovary. Ultrasound Obstet. Gynecol..

[23] Valentin L., Ameye L., Jurkovic D., Metzger U., Lécuru F., Van Huffel S., Timmerman D. (2006). Which extrauterine pelvic masses are difficult to correctly classify as benign or malignant on the basis of ultrasound findings and is there a way of making a correct diagnosis?. Ultrasound Obstet. Gynecol..

[24] Valentin L., Ameye L., Savelli L., Fruscio R., Leone F.P.G., Czekierdowski A., Lissoni A.A., Fischerová D., Guerriero S., Van Holsbeke C. (2011). Adnexal masses difficult to classify as benign or malignant using subjective assessment of gray-scale and Doppler ultrasound findings: Logistic regression models do not help. Ultrasound Obstet. Gynecol..

[25] Marko J., Marko K.I., Pachigolla S.L., Crothers B.A., Mattu R., Wolfman D.J. (2019). Mucinous Neoplasms of the Ovary: Radiologic-Pathologic Correlation. Radiographics.

[26] Pascual A., Guerriero S., Rams N., Juez L., Ajossa S., Graupera B., Hereter L., Cappai A., Pero M., Perniciano M. (2017). Clinical and ultrasound features of benign, borderline, and malignant invasive mucinous ovarian tumors. Eur. J. Gynaecol. Oncol..

[27] Valentin L., Ameye L., Franchi D., Guerriero S., Jurkovic D., Savelli L., Fischerova D., Lissoni A., Van Holsbeke C., Fruscio R. (2012). Risk of malignancy in unilocular cysts: A study of 1148 adnexal masses classified as unilocular cysts at transvaginal ultrasound and review of the literature. Ultrasound Obstet. Gynecol..

[28] Weinberger V., Minář L., Felsinger M., Ovesná P., Bednaříková M., Číhalová M., Jandáková E., Hausnerová J., Chaloupková B., Zikán M. (2018). Brenner tumor of the ovary—Ultrasound features and clinical management of a rare ovarian tumor mimicking ovarian cancer. Ginekol. Pol..

[29] Moro F., Zannoni G.F., Arciuolo D., Pasciuto T., Amoroso S., Mascilini F., Mainenti S., Scambia G., Testa A.C. (2017). Imaging in gynecological disease (11): Clinical and ultrasound features of mucinous ovarian tumors. Ultrasound Obstet. Gynecol..

[30] Moro F., Poma C.B., Zannoni G.F., Urbinati A.V., Pasciuto T., Ludovisi M., Moruzzi M.C., Carinelli S., Franchi D., Scambia G. (2017). Imaging in gynecological disease (12): Clinical and ultrasound features of invasive and non-invasive malignant serous ovarian tumors. Ultrasound Obstet. Gynecol..

[31] Esquivel Villabona A.L., Rodríguez J.N., Ayala N., Buriticá C., Gómez A.C., Velandia A.M., Rodríguez N., Alcázar J.L. (2022). Two-Step Strategy for Optimizing the Preoperative Classification of Adnexal Masses in a University Hospital, Using International Ovarian Tumor Analysis Models: Simple Rules and Assessment of Different NEoplasias in the adneXa Model. J. Ultrasound Med..

[32] Virgilio B.A., Blasis I.D.E., Sladkevicius P., Moro F., Zannoni G.F., Arciuolo D., Mascilini F., Ciccarone F., Timmerman D., Kaijser J. (2019). Imaging in gynecological disease (16): Clinical and ultrasound characteristics of serous cystadenofibromas in adnexa. Ultrasound Obstet. Gynecol..

[33] Goldstein S.R., Timor-Tritsch I.E., Monteagudo A., Monda S., Popiolek D. (2015). Cystadenofibromas: Can transvaginal ultrasound appearance reduce some surgical interventions?. J. Clin. Ultrasound..

[34] Alcázar J.L., Errasti T., Mínguez J.A., Galán M.J., García-Manero M., Ceamanos C. (2001). Sonographic features of ovarian cystadenofibromas: Spectrum of findings. J. Ultrasound Med..

[35] Lu Z., Li B., Gu C. (2019). Outcomes of fertility-sparing surgery for stage II and III serous borderline ovarian tumors. J. Int. Med. Res..

[36] Ludovisi M., Foo X., Mainenti S., Testa A.C., Arora R., Jurkovic D. (2015). Ultrasound diagnosis of serous surface papillary borderline ovarian tumor: A case series with a review of the literature. J. Clin. Ultrasound.

[37] Landolfo C., Valentin L., Franchi D., Van Holsbeke C., Fruscio R., Froyman W., Sladkevicius P., Kaijser J., Ameye L., Bourne T. (2018). Differences in ultrasound features of papillations in unilocular-solid adnexal cysts: A retrospective international multicenter study. Ultrasound Obstet. Gynecol..

[38] Fagotti A., Ludovisi M., De Blasis I., Virgilio B., Di Legge A., Mascilini F., Moruzzi M., Giansiracusa C., Fanfani F., Tropeano G. (2012). The sonographic prediction of invasive carcinoma in unilocular-solid ovarian cysts in premenopausal patients: A pilot study. Hum. Reprod..

[39] Timmerman D., Moerman P., Vergote I. (1995). Meigs’ syndrome with elevated serum CA 125 levels: Two case reports and review of the literature. Gynecol. Oncol..

[40] Hiett A.K., Sonek J.D., Guy M., Reid T.J. (2022). Performance of IOTA Simple Rules, Simple Rules risk assessment, ADNEX model and O-RADS in differentiating between benign and malignant adnexal lesions in North American women. Ultrasound Obstet. Gynecol..

[41] Lai H., Lyu G., Kang Z., Li L., Zhang Y., Huang Y. (2021). Comparison of O-RADS, GI-RADS, and ADNEX for Diagnosis of Adnexal Masses: An External Validation Study Conducted by Junior Sonologists. J. Ultrasound Med..

[42] Chen G.-Y., Hsu T.-F., Chan I.-S., Liu C.-H., Chao W.-T., Shih Y.-C., Jiang L.-Y., Chang Y.-H., Wang P.-H., Chen Y.-J. (2022). Comparison of the O-RADS and ADNEX models regarding malignancy rate and validity in evaluating adnexal lesions. Eur. Radiol..

[43] Basha M.A.A., Metwally M.I., Gamil S.A., Khater H.M., Aly S.A., El Sammak A.A., Zaitoun M.M.A., Khattab E.M., Azmy T.M., Alayouty N.A. (2021). Comparison of O-RADS, GI-RADS, and IOTA simple rules regarding malignancy rate, validity, and reliability for diagnosis of adnexal masses. Eur. Radiol..

[44] Xie W.T., Wang Y.Q., Xiang Z.S., Du Z.S., Huang S.X., Chen Y.J., Tang L.N. (2022). Efficacy of IOTA simple rules, O-RADS, and CA125 to distinguish benign and malignant adnexal masses. J. Ovarian Res..

